# The Effects of Encapsulating Bioactive Irish Honey into Pluronic-Based Thermoresponsive Hydrogels and Potential Application in Soft Tissue Regeneration

**DOI:** 10.3390/gels11030215

**Published:** 2025-03-19

**Authors:** Daniel P. Fitzpatrick, Emma Browne, Carmel Kealey, Damien Brady, Siobhan Kavanagh, Sinead Devery, Noel Gately

**Affiliations:** 1 PRISM Institute, Technological University of the Shannon, N37HD68 Athlone, Co. Westmeath, Ireland; 2 Bioscience Research Institute, Technological University of the Shannon, N37HD68 Athlone, Co. Westmeath, Ireland; a00238871@student.tus.ie (E.B.);; 3 Department of Pharmaceutical Sciences and Biotechnology, Technological University of the Shannon, N37HD68 Athlone, Co. Westmeath, Ireland; 4 Applied Polymer Technologies (APT), Technological University of the Shannon, N37HD6 Athlone, Co. Westmeath, Ireland; 5 Department of Science and Health, South East Technological University, R93V960 Carlow, Co. Carlow, Ireland

**Keywords:** antimicrobial, bioresorbable, drug delivery, honey, hydrogel, wound regeneration

## Abstract

Honey has been recognised for centuries for its potential therapeutic properties, and its application in wound healing has gained attention due to its antimicrobial, anti-inflammatory, and regenerative properties. With the rapid increase in multidrug resistance, there is a need for new or alternative approaches to traditional antibiotics. This paper focuses on the physicochemical changes that occur when formulating honey into Pluronic F127 hydrogels. The manual incorporation of honey, irrespective of quality type, presented the amelioration of Pluronic’s capacity to undergo sol–gel transitions, as investigated by parallel plate rheology. This novel finding was attributed to the formation of fractal aggregates via the hydrogen-bonding-induced irreversible aggregation of honey–PF127 micelles, which subsequently dominate the entire hydrogel system to form a gel. The hydrogen bonding of micelles was identified through Attenuated Total Reflectance Fourier-Transform Infrared Spectroscopy (ATR-FTIR), Differential Scanning Calorimetry (DSC), and Dynamic Light Scattering (DLS). This is the first known study to provide physicochemical insight into the effects that honey incorporation has on the thermogelation capacity of Pluronic F127 hydrogels for downstream dermal wound applications.

## 1. Introduction

The management of complex dermal wounds such as deep tissue injuries, surgical wounds, and pressure ulcers can be challenging and requires a multifaceted treatment approach. The goals of wound management include reducing pain and discomfort, preventing reinfection, and promoting tissue regeneration. The intricate process of wound healing arises from the multifaceted interaction of genetic, immunological, environmental, and microbial factors, all of which influence tissue regeneration [1]. One approach to wound management for complex dermal wounds is the use of advanced wound dressings. These dressings are formulated to maintain a moist wound environment, aiding in pain relief, inflammation reduction, infection prevention, and enhanced tissue regeneration. Advanced wound dressings can also be used to manage wound exudate, which is the fluid that can leak from the wound bed and contribute to tissue damage and infection. There are several types of advanced wound dressings available for the management of complex dermal wounds, including foam dressings, hydrocolloid dressings, and silver dressings. Absorbent foam dressings absorb and help manage wound exudate, while hydrocolloid dressings provide a protective barrier against external contaminants and can be used on wounds with moderate to heavy exudate [2,3,4].

Therapeutically active honey dressings have antimicrobial, antioxidant, and immunomodulatory effects and have been shown to improve wound healing, including in chronic diabetic wounds [5,6] and pressure ulcers [7]. The high-sugar, acidic nature of honey, along with the production of hydrogen peroxide and the synergistic effect of phenolic compounds [8] present in the honey, provides a wide-ranging antimicrobial agent effective against common wound pathogens and biofilms such as *Staphylococcus aureus*, *Staphylococcus epidermidis*, *Pseudomonas aeruginosa*, and *Escherichia coli* [9,10,11]. Phenolic compounds and vitamins contribute to the antioxidant properties of honey [12] as well as directly inhibiting and/or stimulating cell antioxidants [13,14]. Further to this, many compounds identified in honey, such as exosome-like vesicles [15], 5-hydroxymethylfufural [16], arabinogalactan [17], bee-defensin 1 [18], and Major Royal Jelly Proteins [19], can inhibit or stimulate cytokine production in cells, thus affecting cell signalling, migration, and inflammatory pathways. Honey has also shown effects on cell migration, improving wound healing rates as well as improving tissue regeneration and reducing scarring [20].

While innate honey has been applied to wounds in the past, this can have its own complications due to the cytotoxicity of high concentrations of honey. Additionally, innate honey that is not of medical grade may harbour microbial contamination [21], and its innate viscous nature could further complicate complex wounds. The incorporation of honey into medical scaffolds and hydrogel systems facilitates a means to overcome these barriers and provide efficient wound regeneration. Honey is often applied with sterile spatulas to the wound bed, followed by a sterile, absorbent dressing pad, cast padding as a secondary layer, gauze as tertiary layer, and a final adhesive outer layer [22]. This is dependent on the wound type as well as whether debridement is required. Ideally, a medical-grade (sterilised) honey should be used whereby the compounds in the honey are not denatured due to heat [23] or light [24], but any microorganism contamination is removed. Easy application methods such as squeezable tubes reduce the risk of cross-contamination.

The high osmolarity of honey that would absorb the wound exudate presents an inverse issue as it also facilitates the honey to leak out of the wound bed [25]. In addition to this, a recent review conducted by Nezhad-Mokhtari et al. (2021) noted that the lack of sustained release rates and the toxicity of high concentrations of honey over time are parameters that require attention to improve honey-based tissue engineering scaffolds [26]. The most widely studied honey-incorporated delivery systems currently under investigation include cryogels, hydrogels, and electrospun fibres [27] such as carboxymethyl cellulose pastes [28], chitosan/alginate sponges [29], polyacrylic acid/chitosan [30], alginate gels [31,32], xanthum gum [33], Carbopol 940/chitosan [34], chitosan/PVA film [35], and PVP/agar/PEG emulgels [36].

Pluronic hydrogel systems are a class of thermosensitive surfactants that have attracted significant attention in the field of tissue engineering and regenerative medicine [37]. These hydrogels consist of block copolymers of poly(ethylene oxide) (PEO) and poly(propylene oxide) (PPO) that are capable of forming a gel at body temperature [38]. Pluronic hydrogel systems offer several advantages over other hydrogel systems, including their ease of preparation, versatility, biocompatibility, and tuneable properties [37]. Pluronic hydrogels can be easily prepared by dissolving the copolymer in an aqueous solution, and the gelation process is triggered by a change in temperature, which can be controlled by the formulation of the hydrogel. Understanding how the material changes over time between storage conditions and the point of clinical application enables the user to determine at which time point along the sol–gel transition period is best for patient–patient variation in wound care [39]. The mechanism through which surfactants such as Pluronic can support wound regeneration is attributed to several facets of the innate material, such as aiding wound cleansing, suppressing protein aggregation and denaturisation, biocompatibility, and exerting antimicrobial activity. Surfactants warrant autolytic debridement through the degradation of collagen debris by altering the enzymatic activity of matrix metalloproteinases (MMPs) [40]. One advantage of Pluronic hydrogels in wound healing is the high water content, which can warrant the retention of a moist wound environment, known to promote faster and more effective wound healing. The retention of a moist environment inhibits the formation of scabs, which can impede the healing process [38,41]. The incorporation of honey for achieving higher-order structures through their unique composition of sugars and polyols presents an intriguing mechanism as it avoids the use of chemical cross-linking agents, initiators, and catalysts. Hydrogels have also been shown to provide moist environments with the excellent fluid absorbance important for wounds due to initial fluid exudate, but lead to the eventual drying of wounds. Choosing a suitable gel formation is important to ensure the easy application of the honey, as well as the complete incorporation and suitable diffusion of the honey to the wound bed.

This is the first known study to provide an insight into the effects of the incorporation of different honey varieties on the thermogelation capacity of Pluronic F127 hydrogels for downstream dermal wound applications.

## 2. Results and Discussion

The incorporation of honey into a stable hydrogel of Pluronic F127 was initially attempted via the cold method; however, it was observed that low-concentration Pluronic hydrogels (<25%) were not capable of forming stable systems, as the honey and Pluronic would separate in response to temperature. At concentrations of Pluronic F127 greater than 25%, it was noticed that the bioactive hydrogel became stiff even at low temperature, indicative of a change in the reversible thermoresponsive nature of innate Pluronic F127. Honey is complex, with compounds that are light- and heat-sensitive [42,43]. Phenolic compounds, which contribute to the antioxidant and immunomodulatory properties of the honey, are extremely light-sensitive. Proteins such as bee defensin-1 or glucose oxidase for hydrogen peroxide production may become denatured if heated over 37 °C. Because of this, honey that is not stored in the dark or that is heated above 37 °C will lose medicinal activity. Thus, sterilising honey with UV light or heat is contraindicated. Due to the highly viscous nature of honey, sterile filtering is difficult to carry out unless the honey is first diluted to concentrations as low as 20%.

### 2.1. Attenuated Total Reflectance Fourier-Transform Infrared Spectroscopy (ATR-FTIR)

**Figure 1:**
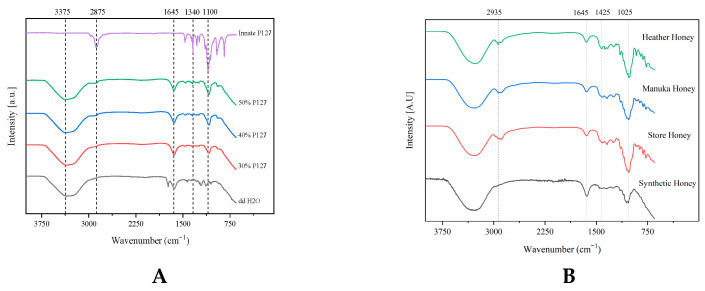
FTIR spectra of hydrated Pluronic F127 hydrogels (**A**) and innate honey samples (**B**) utilised in the development of bioactive PF127 hydrogels.

The FTIR spectra of innate Pluronic F127 shown in Figure 1A were characterised by principal absorption peaks at 2875 cm^−1^ (C–H stretching vibrations), 1340 cm^−1^ (O–H bend), 1278 cm^−1^ and 1240 cm^−1^ (C–O–C stretches), and 1100 cm^−1^ (C–O stretching vibrations). Although the intensity of these peaks was dampened after the polymer was hydrated with double-distilled water, all peaks were retained across all formulations once hydrated with double-distilled water. Pluronic hydrogels displayed prominent absorption peaks at 3300 cm^−1^ (OH stretching band) and 1640 cm^−1^, which are attributed to polymer hydration. At 3300 cm^−1^, the molecular environment of the hydrogen-bonded molecules was deemed heterogeneous across all formulations, as band broadening was observed [44]. As the polymer concentration increases, the intensity of C–H stretching vibrations from the Pluronic material increases.

As shown in Figure 1B, the innate honey samples presented several distinctive characteristic peaks that align with the literature [45,46]. The water-based nature of these formulations presented as a large broad peak at 3375 cm^−1^, representing the O–H stretching of water and honey-derived carboxylic acids. The C–H stretching of carbohydrates and the NH_3_ stretching of free amino acids was identified as a broad peak at 2935 cm^−1^. Water deformation was observed at 1645 cm^−1^, attributed to the O–H stretching/bending of water and C=O stretching of carbohydrates. This carbohydrate deformation was presented as an array of peaks ranging from 1540 to 1175 cm^−1^ and represented the O–H stretching/bending, C=O and C–H stretching of carbohydrates, and C=O stretching of associated ketones. Stretching of the C–O and C–C of carbohydrates and ring vibrations corresponded to peaks with lower wavenumbers ranging from 1175 to 940 cm^−1^. These peaks are predominantly from carbohydrates than other constituents of honey.

The store honey and the synthetic honey displayed characteristic spectra of sugar syrups and it was not possible to differentiate between the individual constituents with the applied method. As these samples consisted of prominent sugars in honey, several characteristic adsorption peaks of honey constituents at 2900 cm^−1^, 1500–1100 cm^−1^, and below 900 cm^−1^ were not present as these peaks are attributed to the vibrational stretching/bending of carbohydrates and other constituents of honey.

**Figure 2:**
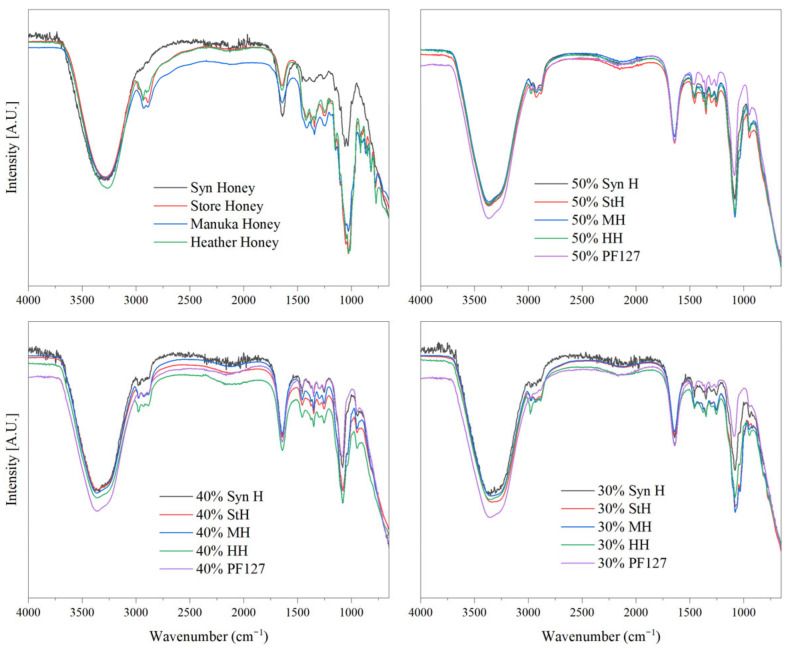
FTIR spectra of innate PF127 hydrogels and bioactive hydrogels of various encapsulated honey types.

In contrast, heather honey could be differentiated from other honey types with three distinctive minor peaks within the 1500–1250 cm^−1^ wavenumber range. These distinctive peaks may correspond to the presence of 2-methylbutyric acid and isophorone, specific chemical markers for assessing the botanical and geographical origin of heather honey [47,48]. Similarly, manuka honey displayed the characteristic peaks of honey and could be distinguished through the vibration bands of carbohydrates ranging from 1500 cm^−1^ to 700 cm^−1^ [46,49,50]. The incorporation of each honey into the hydrogel matrix was deemed compatible with the retention of all characteristic peaks of PF127 and the respective honey peaks shown in the FTIR spectra of Figure 2. Although compatible, several minor bathochromic and hypsochromic shifts were observed in the region of 1449–1023 cm^−1^ and were attributed to hydrogen bonding interactions between the hydroxyl groups of honey moieties and the micelles of Pluronic F127.

**Figure 3:**
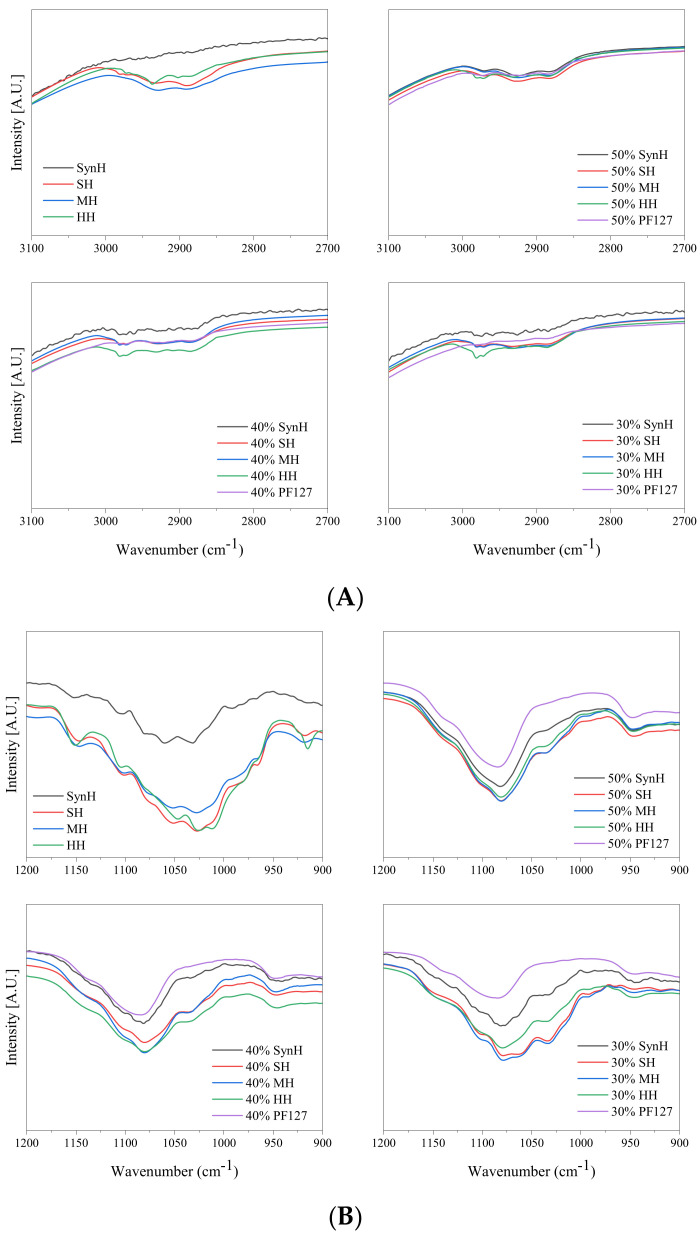
FTIR spectra of molecular interactions at ~2950 cm^−1^ (**A**) and ~1050 cm^−1^ (**B**) as a consequence of incorporating the respective honey into PF127 hydrogel systems ranging from 30 to 50%.

Intermolecular interactions between the polymer chains of PF127 hydrogels and the encapsulated honey were identified and are displayed in Figure 3. The FTIR spectra of bioactive hydrogels were observed to have peak interactions between 3000 cm^−1^ and 2950 cm^−1^, representing OH stretching vibration, i.e., υ_O–H_ and intramolecular hydrogen bonding [51]. These interactions are attributed to hydrogen bonding between the hydroxyl group of Pluronic’s polyethylene oxide moiety and those of honey constituents. The complex composition of each honey type makes it difficult to specify which components of honey cause this effect. Heather honey was noted as having the greatest degree of interaction as the peaks at 2980 cm^−1^ were retained as the polymer concentration increased. Defining the strength of hydrogen bonding can be denoted through the shifting of the bending OH vibrational frequency of water at ~1650 cm^−1^, as it is sensitive to intra- and intermolecular hydrogen bonding groups [52]. The hydrogen bonding of PF127–honey can be defined as “weak” as the bending OH vibrational frequency of water did not shift from 1640 cm^−1^ in response to varied honey concentrations.

### 2.2. Differential Scanning Calorimetry (DSC)

**Figure 4:**
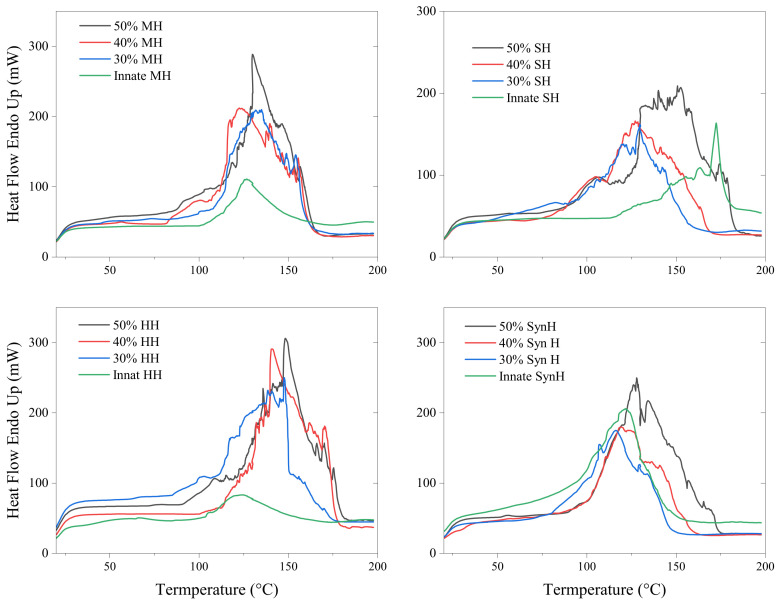
DSC thermograms of bioactive hydrogels to evaluate the plasticisation–anti-plasticisation capacity of honey types on Pluronic F127.

All samples displayed wide and intense endothermic peaks ranging from an onset temperature of 100–120 °C to an end temperature of between 180 and 220 °C corresponding to the melting of all sugars in the samples (Figure 4) [53]. Irrespective of honey type, the degree of plasticisation increased with polymer concentration. One mechanism through which honey can act as a plasticiser is by forming hydrogen bonds with moieties of the polymer network. These hydrogen bonds can increase the flexibility of the hydrogel system and improve mechanical properties including elasticity and tensile strength [54]. Interactions with the hydrophobic tails of the surfactant can affect the molecular packing and orientation of the micelle, which can result in changes in the size and shape of the Pluronic micelle as well as its stability [55,56]. Hydrogen bonding can dictate the arrangement of molecules in the solid state. Molecules that engage in hydrogen bonding tend to organise themselves in specific orientations to optimise the formation of these bonds. This arrangement leads to a more ordered, closely packed structure, which, in turn, contributes to a higher melting temperature.

**Figure 5:**
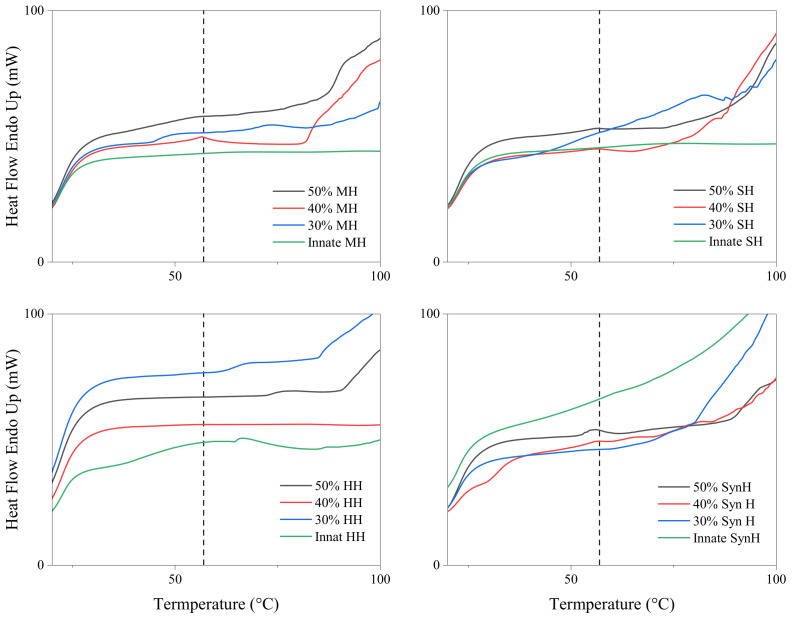
DSC thermograms of bioactive hydrogels to evaluate the plasticisation–anti-plasticisation capacity of honey types on the melt temperature peak of Pluronic F127.

The thermograph of innate Pluronic F127 hydrogel displayed a single endothermic peak at 57 °C, which is within the range of the innate material (52–57 °C) [57,58]. As the samples were assessed in their hydrogel state, the inclusion of water is attributed to prolonging the crystallinity of the polymer; thus, the endothermic peak was detected at the higher end of this melt range. The primary constituents of honey have been reported by Cordella et al. (2002) [53] as sugars and water, while proteins, pigments, aromatic compounds, and various volatiles make up the remaining fraction [59]. The variations observed in the honey thermographs is attributed to honey’s lack of a distinct and well-defined melting point, variations in the respective complex composition, and thermal processing. The melt peak of Pluronic F127 (58 °C) can be identified in several samples in Figure 5, presenting as broad extending peaks in the 30% formulations due to the changes in water content. As the polymer concentration increases, the Pluronic peak presents either as a single peak, indicating heterogeneity, or the peak is not present at all, indicating homogeneity throughout the bioactive hydrogel. The presence/loss of Pluronic’s melt peak may be attributed to the homogeneity between the honey constituents and the polymer network. The general thermal response of all honey types was comparable to those previously published by Cordella et al. (2002) [53]. The relatively weak endothermic phenomenon associated with the melting of the starch/sugar/water complex or polymorphs of sugars is presented in Figure 5.

### 2.3. Rheological Evaluation

#### 2.3.1. Amplitude Sweep

The deformation response of Pluronic hydrogels was assessed across the oscillation strain range from 0.01 to 100 rads/s. In accordance with the ISO 6721-10 and EN/DIN EN 14770 standards, it was determined that a 1% strain was sufficient for subsequent testing as the polymeric material deformed by less than 5% [60,61]. As all formulations consisted predominantly of PF127, all bioactive hydrogels were also subjected to a 1% strain for subsequent testing.

#### 2.3.2. Frequency Sweep

**Figure 6:**
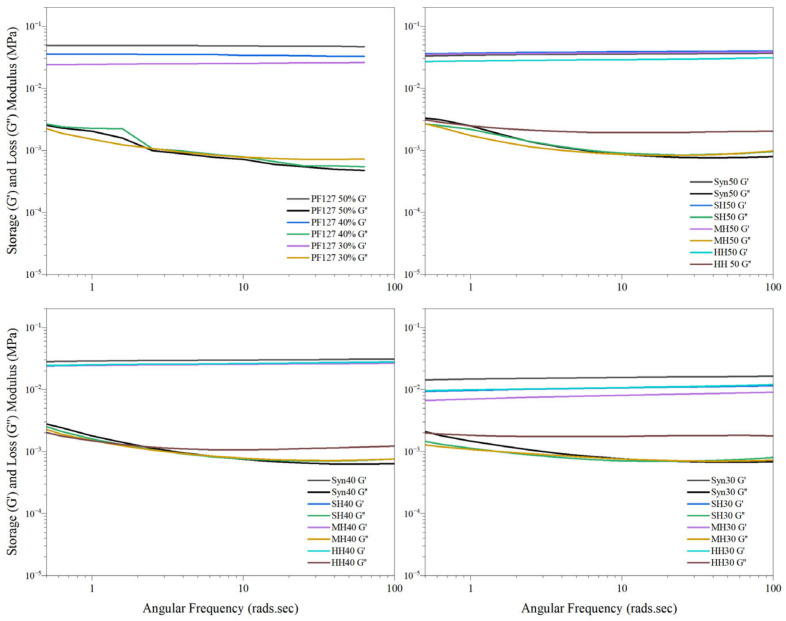
Frequency sweeps of PF127 innate hydrogels and bioactive hydrogels of respective honey types.

A hydrogel’s unique structure enables the exhibition of both elastic and viscous responses to applied frequencies. It was important to assess all material responses across a range of frequencies because many materials exhibit complex and frequency-dependent mechanical behaviours. All innate Pluronic formulations displayed an overall dominance of the storage modulus with a weak dependence on the frequency. The stability of all bioactive formulations was observed, as shown in Figure 6, as G′ remained greater than G″ throughout, and this implied that all samples had undergone complete sol–gel transition at 37 °C with no indication of relaxation throughout the test. These results are comparable with recent results published on Pluronic formulations ranging from 15 to 30% [62]. The storage modulus of innate PF127 hydrogels behaved in a concentration-dependant manner. The instability of the gel matrix of PF127 at lower frequency may provide insight into why some formulations have the capacity to become heterogeneous in response to stressors such as strain or temperature.

The incorporation of synthetic honey into the matrix of the PF127 network retained the greatest comparable storage modulus to innate PF127 with respect to polymer concentration, as shown in Figure 6. This was attributed to the water content and a high degree of hydrogen bonding between the monosaccharides and polymer chains. In contrast, at the lower polymer concentration of 30% (*w*/*w*), manuka, heather, and store honey displayed the lowest storage modulus, indicating that honey constituents other than the primary sugars may be inducing a higher degree of heterogeneity within the respective hydrogel systems. The storage moduli of HH, MH, and SH hydrogels increased incrementally with respect to polymer concentration, attributed to greater homogeneity as the polymer concentration increased.

The varied storage moduli of bioactive hydrogels may be attributed to a complex relationship denoted by Anopchenko et al. (2006) [63] between the plasticisation and anti-plasticisation capacity of honey constituents. Common substances that exhibit anti-plasticisation effects include water, polyols, and various mono- and disaccharides, though this behaviour is highly dependent on specific temperature and concentration conditions [63]. A Tukey comparison of all formulations deemed that the incorporation of honey significantly (<0.05) reduced the storage modulus of innate Pluronic hydrogels, with the exception of the 50% MH and the 50% SH formulations.

**Figure 7:**
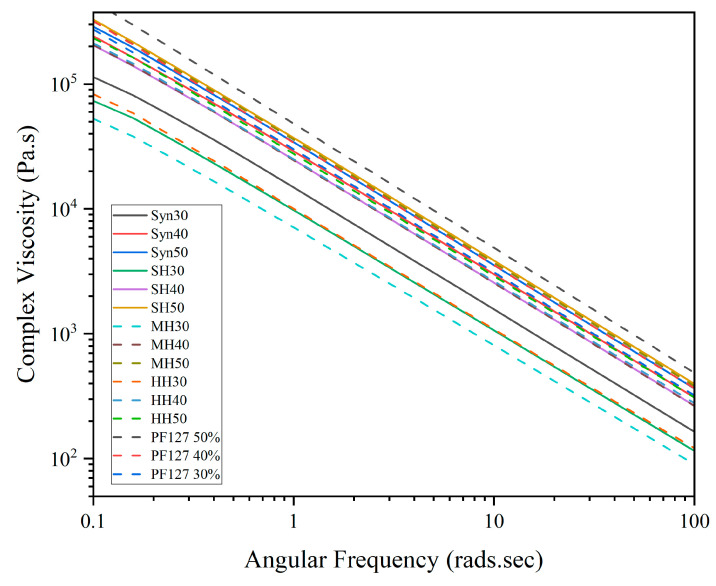
Complex viscosity of bioactive hydrogels in response to increasing honey concentrations.

For medical applications, hydrogels may be subjected to various types of stresses and deformations, including shear stress, compression, and extension, adjacent to surgical procedures such as injection, spreading, smearing, and manipulation. The complex viscosity of hydrogel formulations can help to predict how they will behave under these different conditions, and it can also provide insight into the potential for the material to undergo unwanted deformation or flow characteristics. As shown in Figure 7, the complex viscosity of all bioactive and innate formulations was observed to decrease as the angular frequency increased, confirming that all samples behaved as pseudoplastic fluids with shear-thinning behaviour, whereby the hydrogels become less viscous and more fluid-like when subjected to shear stress or deformation. For downstream biomedical applications, the shear-thinning behaviour of hydrogel formulations is favourable, as this behaviour can allow the fluid to be easily injected or applied to a specific site while still maintaining its structural integrity and stability over time [64].

#### 2.3.3. Temperature Sweep

**Figure 8:**
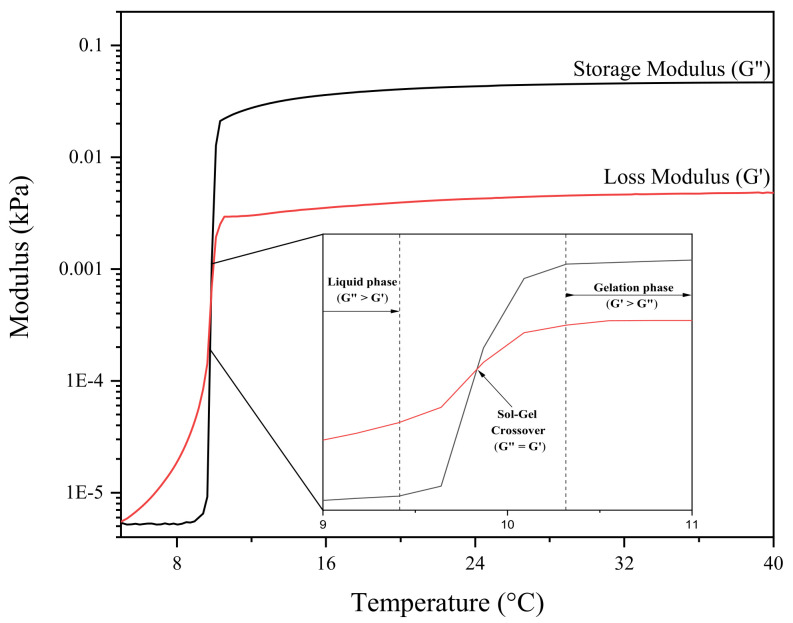
Temperature sweep of Pluronic 50% depicting storage modulus (G′) and loss modulus (G″) changes over temperature ranging from 4 to 40 °C. The liquid phase of the sample is denoted within the region (G″ > G′), the sol–gel crossover point is denoted as (G′ = G″), and the following region represents the sample in the gel phase, where (G′ > G″).

The sol–gel transition temperature was identified as the first recorded temperature at which the storage modulus (G′) surpassed the loss modulus (G″), marking the shift from a predominantly viscous state (G′ < G″) to a predominantly elastic state (G′ > G″), as illustrated in Figure 8 [65,66]. The thermoresponsive behaviour of Pluronic hydrogels exhibited an inverse relationship with polymer concentration, where an increase in polymer concentration corresponded to a decrease in gelation temperature. Understanding how the material changes in response to the incorporation of bioactive additives such as honey allows for the selection of ideal properties for clinical applications, which enables the user to determine at which time point along the sol–gel transition period is best for patient–patient variation in wound care.

The rheological behaviour of honey is influenced by various factors, including its constituent composition and ambient temperature. As the temperature increases, intermolecular forces weaken, leading to a reduction in viscosity. This occurs due to a decrease in molecular friction and hydrodynamic resistance, allowing the honey to flow more easily at higher temperatures [67,68,69,70]. The incorporation of honey, irrespective of type, presented the amelioration of Pluronic’s capacity to undergo sol–gel transitions. This novel finding is attributed to the formation of fractal aggregates via the hydrogen-bonding-induced irreversible aggregation of honey–PF127 micelles, which subsequently dominate the entire hydrogel system to form a gel.

**Figure 9:**
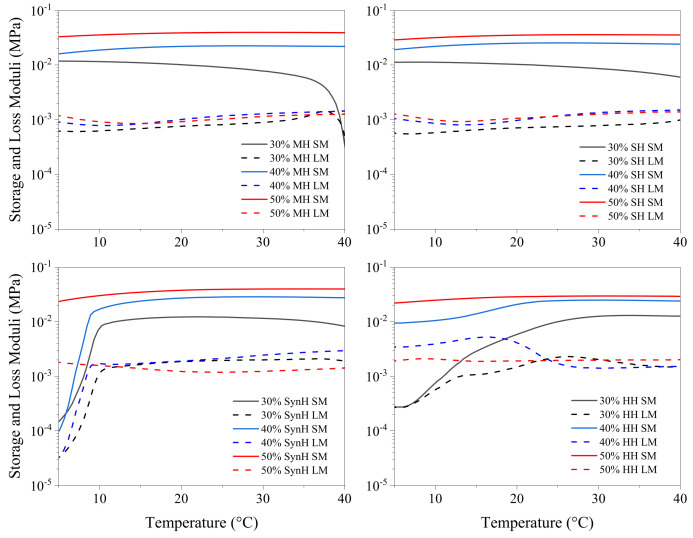
Temperature sweep of bioactive hydrogels ranging from 30 to 50% Pluronic concentration, depicting how the storage modulus (G′) and loss modulus (G″) change over a temperature ranging from 4 to 40 °C.

Competition between honey components and the hydrophilic moiety of Pluronic F127 may lead to a reduction in the effective concentration of surfactants in the solution, resulting in a decrease in the critical micelle concentration (CMC), thus making it easier for Pluronic molecules to form micelle structures in the hydrogel system [71]. Heterogeneity within the respective honey hydrogel networks may contribute to the varied response as the temperature increases. Temperature increases result in the reduction of intermolecular forces and viscosity; hence, the honey’s viscosity is reduced due to decreased hydrodynamic forces and molecular friction. As shown in Figure 9, the dominant viscous nature of honey was observed in the 30% (*w*/*w*) formulations of manuka and store honey as the storage moduli declined as temperature increased. All other formulations displayed predominant elastic (G′ > G″) behaviour over the temperature range; however, the synthetic and heather honey presented a thermoresponsive effect as the moduli increased incrementally but retained the elastic (G′ > G″) behaviour.

### 2.4. Dynamic Light Scattering (DLS)

The unforeseen changes in the rheological properties warranted greater investigation into the variation of Pluronic’s responsive behaviour in response to the incorporation of honey. The size distribution of innate honey samples and the incorporated hydrogels are outlined in Table 1. The requirement to dilute (1:10) all samples for DLS decreased the assessment concentration of PF127 to 2–5%. In contrast to the results reported by Chung et al. (2020), innate PF127 samples presented a unimodal distribution peak across the 10^0^–10^4^ size range with the hydrodynamic radii of 22.5–24.52 nm with respect to concentration [72].

Monodispersed polymer formulations typically have a polydispersity index (PDI) value of 0.1. A PDI greater than 1 signifies polydispersion as the Mn is lower than the Mw. The polydispersity index of each innate formulation indicates that the formulations are homogeneous. As the polymer concentration increases, the PDI value typically decreases for monofloral honey samples (HH and MH), indicating a higher degree of homogeneity within the hydrogel system, whereas monosaccharide samples (SH and SynH) retain constant PDI values irrespective of the polymer concentration and retain a high order of homogeneity.

Given the varied composition between the honey samples, the incorporation of each honey was observed to interact with the PF127 micelle structure through different mechanisms. The hydrodynamic radii results shown in Table 1 show that synthetic honey did not induce major changes to the micelle structure across all polymer concentrations, whereas all other honey samples were observed to aggregate the micelle network and alter the micelle in an inverse relationship to the polymer concentration. Store honey and manuka honey displayed the same behaviour, in that large micelles of ~130–150 nm were formed when the polymer concentration was <50%, but sharply declined in size to 32–59 nm at the 50% (*w*/*w*) polymer concentration. Heather honey displayed an inverse linear regression between polymer concentration and micelle radii, indicating a higher degree of integration into the micelle structure than the other honey samples. This aligns with the PDI results, as greater heterogeneity was observed.

**Figure 10:**
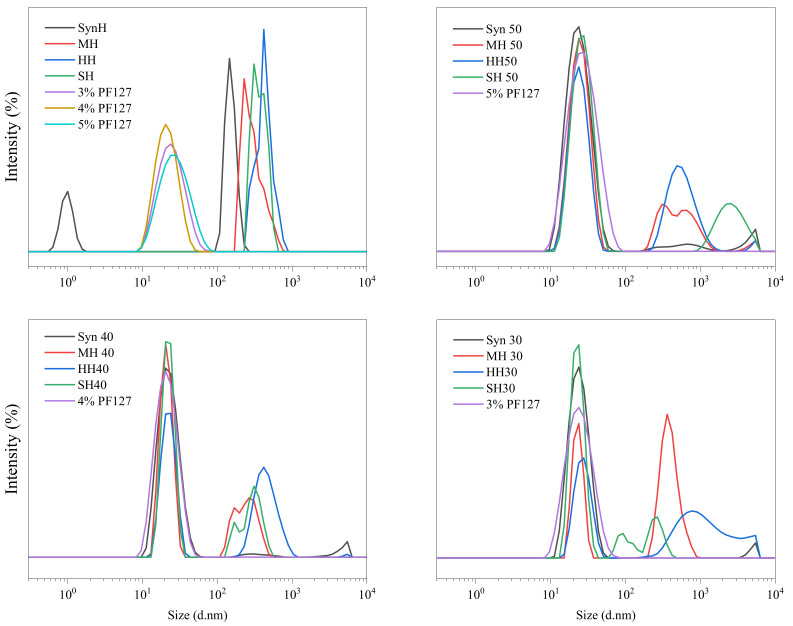
Hydrodynamic radii distribution of PF127 innate hydrogels and aggregates formed by Pluronic F-127 and encapsulated honey types. Distribution range depicts the average of five independent samples for each formulation.

The influence of water content on manuka honey disassociates as the polymer concentration increases, attributed to peak separation into two smaller peaks at the 10^2^–10^3^ size range. Downshifting of the peak size can be attributed to stronger interactions between the polymer chains and the honey moieties. Heather honey presented the greatest stability with the retention of a single peak as the polymer concentration increased. In Figure 10, the broadening of large aggregates at 30% (*w*/*w*) may be due to hydrogen bonding as a consequence of the higher water content within the system in relation to polymer. Manuka and heather honey were observed to have the greatest degree of aggregation with the formation of larger micelles ranging from 10^2^ to 10^4^.

In contrast, store honey presented a higher degree of aggregation as the polymer concentration increased, as shown in Figure 10. As the polymer concentration increases, the two minor peaks integrate and form a large broad agglomerate peak at the 50% (*w*/*w*) formulation. Synthetic honey presented integration of the honey associated peaks into the Pluronic peak, as denoted by a reduction in intensity. The formation of a minor peak within the 10^3^–10^4^ size range was observed as the polymer concentration increased with bimodal distribution at 30% (*w*/*w*) and trimodal distribution as the polymer concentration increased. The samples retain a monodispersed population of particles as no major shift in PDI was observed. The degree of aggregation did, however, occur in a concentration-dependent manner, noting that the addition of honey induced a higher degree of heterogeneity of the hydrogel system, as shown in Figure 10, where the PDI value decreases from 0.284 ± 0.013 to 0.242 ± 0.021.

## 3. Conclusions

This study systematically explored the incorporation of four distinct honey varieties—synthetic honey (representing primary sugar composition), store honey (a highly liquid commercial honey), Manuka honey (a standardised and quality-assured medical-grade honey), and Irish heather honey (a thixotropic honey with unique rheological properties)—into Pluronic F127 hydrogels. The findings provide insights into how honey composition influences the structural, rheological, and mechanical properties of these bioactive hydrogels.

Despite the natural variability in honey composition, key trends were identified that can inform the development of reproducible Pluronic-based hydrogels with minimal dependence on honey type. FTIR analysis indicated weak hydrogen bonding between honey and Pluronic F127, suggesting that honey does not significantly disrupt the micellar network. However, increasing polymer concentration led to enhanced plasticisation effects, likely due to hydrogen bonding between honey moieties and the polymer, which improved hydrogel flexibility and mechanical integrity.

The storage modulus varied across honey types and polymer concentrations. Synthetic honey retained the most comparable storage modulus to native Pluronic, suggesting that formulations primarily reliant on the sugar content of honey exhibit greater stability. In contrast, manuka, heather, and store honey led to greater heterogeneity at lower polymer concentrations (30% *w*/*w*), indicating that secondary bioactive components beyond sugars influence hydrogel properties. However, at higher polymer concentrations (>40% *w*/*w*), these differences diminished, suggesting that greater polymer content improves formulation consistency across honey types.

Rheological analysis confirmed that all hydrogels displayed pseudoplastic (shear-thinning) behaviour, meaning that their viscosity decreased under applied stress. This is a desirable property for biomedical applications, facilitating easy injection or topical application while maintaining structural integrity post-administration. Additionally, temperature-dependent viscosity changes aligned with the known behaviour of honey, where increasing temperature reduced intermolecular forces and lowered viscosity.

A key finding was that honey incorporation enhanced the sol–gel transition behaviour of Pluronic F127, potentially improving gel stability and providing a controlled-release platform for bioactive components. These effects indicate that formulations with higher polymer concentrations and synthetic or highly standardised honey types may offer greater reproducibility.

Overall, this study demonstrates that honey composition significantly influences the mechanical and structural properties of Pluronic F127 hydrogels. To ensure formulation consistency, higher polymer concentrations (>40% *w*/*w*) and honey types with well-defined bioactive markers are recommended. These findings provide a foundation for optimising honey-based hydrogels for biomedical applications, with future research needed to explore biological effects, cytotoxicity, and antimicrobial properties while refining honey selection based on bioactive standardisation.

## 4. Materials and Methods

### 4.1. Materials

A manuka honey (MGO 250+) (Manuka Health, Auckland, New Zealand) (MH) and a store honey (squeezable clear honey from Highgate Fayre) (SH) were purchased as representatives of current market honey. Artificial/synthetic lab honey (SynH) was prepared in distilled water with final concentrations of 40.5% fructose (Sigma-Aldrich, Dublin, Ireland), 33.5% glucose (Sigma-Aldrich, Dublin, Ireland), 7.5% maltose (Sigma-Aldrich, Dublin, Ireland), and 1.5% sucrose (Sigma-Aldrich, Dublin, Ireland). Irish heather honey (*Calluna vulgaris*) was generously provided from a cohort of Irish honey producers. Pluronic F127 (PF127) was purchased from Sigma-Aldrich Ireland Ltd. (Arklow, Wicklow, Ireland). Ultrapure water was obtained from a Barnstead Smart Pure Pro water purification system (Thermo Fisher Scientific, Waltham, WA, USA).

### 4.2. Methodologies

#### 4.2.1. Hydrogel Preparation and API Integration

High-concentration Pluronic F127 hydrogels ranging from 50 to 60% (*w*/*v*) were prepared using the cold method described by Schmolka with an adaptation to account for sterilisation [73]. However, the sterilisation process is performed after hydrogel formulation, which introduces several potential concerns, including moisture loss, polymer degradation, free radical formation, and changes in rheological behaviour [74,75,76]. This approach builds upon the method previously described by Fitzpatrick et al. [77], who investigated the influence of sterilisation on Pluronic-based systems. To mitigate these issues, Pluronic F127 was sterilised prior to hydrogel preparation. The polymer was autoclaved in a 100 mL Duran flask, positioned horizontally, at 121 °C for 15 min. To enhance solubility, the flask was removed while still at approximately 80 °C, allowing the material to distribute more evenly across the inner surface. Once transferred into a grade-II biosafety cabinet, the appropriate volume of sterile double-distilled water (ddH_2_O) was introduced. The flask was then placed on ice and periodically shaken to ensure complete solubilisation of the sterile Pluronic F127.

#### 4.2.2. Preparation of Bioactive Hydrogel

Sterilised innate Pluronic hydrogels were produced after an adapted steam sterilisation method [77]. Honey was stored at 4 °C in the dark to prevent the light and heat degradation of sensitive compounds [42,43]. Prior to inclusion into the Pluronic hydrogels, the honey was not sterilised. Bioactive hydrogels were prepared by physically mixing high-concentration PF127 hydrogels with weighed amounts of honey to achieve a final polymer concentration range of 30–50% (*w*/*w*). The 50% (*w*/*w*) hydrogel was prepared by combining a pre-weighed amount of 60% (*w*/*w*) PF127 hydrogel with the corresponding weight of honey. Similarly, the 40% (*w*/*w*) and 30% (*w*/*w*) formulations were prepared by diluting pre-weighed 50% (*w*/*w*) PF127 hydrogel with the appropriate weight of honey to reach the desired final polymer concentration, as detailed in Table 2. All formulations were physically mixed in a small mortar and pestle and stored at room temperature until required for analysis.

#### 4.2.3. Attenuated Total Reflectance Fourier-Transform Infrared Spectroscopy

ATR-FTIR spectroscopy was conducted according to the method described by [77] with the following parameters. The spectra of the samples were performed on an ATR-FTIR spectrophotometer (Perkin Elmer Spectrum One with a universal ATR sampling accessory). Samples were scanned between 650 and 4000 cm^−1^, with 4 scans per sample on average, and a fixed universal compression force of 80 N. Further analyses were carried out using Origin software (2024b).

#### 4.2.4. Differential Scanning Calorimetry

The thermal properties of innate honey, Pluronic hydrogels, and bioactive hydrogels (~15 mg) were analysed in duplicate using a PerkinElmer Pyris 1 DSC (PerkinElmer Inc., Waltham, MA, USA) as adapted from a previous publication [77]. A single heating and cooling cycle was conducted at a scanning rate of 10 °C min^−1^. The resulting data were processed using Pyris data analysis software Version 11 and further refined in Origin for graphical representation and analysis.

#### 4.2.5. Rheological Evaluation

The rheological characteristics of all formulations were assessed using a DHR20 rheometer (TA Instruments, New Castle, DE, USA) equipped with a refrigerated cooling system (RCS120), as adapted from a previous publication [77]. Data acquisition and analysis were performed using TRIOS software (v5.1.1.46572). A 50 mm stainless steel plate was used in conjunction with a Peltier plate to maintain precise temperature control. Samples were introduced in their cold state using either a spatula or a 3 mL Pasteur pipette for 1.0 mL volumes. To ensure environmental stability and eliminate artefacts from loading stress, a 60 s soak period was observed prior to measurement. Following this, quintuplet amplitude sweeps were conducted to establish the linear viscoelastic region (LVER), which guided the selection of an appropriate oscillation stress (1%) for subsequent measurements. The storage modulus (G′) was recorded as samples underwent oscillatory strain ranging from 0.01% to 100% at a constant frequency of 6.28 rad/s. The LVER for each sample was defined as the range where the storage modulus varied by no more than 5% between measurements, following standards ISO 6721-10 and EN/DIN EN 1477 [60,61].

##### Frequency Sweep

Oscillation frequency sweeps were performed at 37 °C, covering an angular frequency range from 0.01 to 100 rad/s, while maintaining a constant strain of 1%. Measurements were recorded in triplicate.

##### Temperature Sweep

Temperature-dependent rheological changes were assessed by conducting temperature sweeps over a range of 4 °C to 40 °C under a constant strain of 1%, as determined in the amplitude sweep. Measurements were recorded in triplicate.

#### 4.2.6. Dynamic Light Scattering

The hydrodynamic radii of both innate and bioactive hydrogels were determined using non-invasive backscatter (NIBS) dynamic light scattering (DLS) with a static scattering angle of 173° (Zetasizer Pro; Malvern Instruments, Worcestershire, UK) at 20 °C (room temperature) adapted from [77]. Formulations were diluted with sterile water 1:10 prior to DLS processing. Samples were not filtered through 0.45 µm microfilters. Solutions were processed in quintuplicate and data processing was conducted using ZS Xplorer software Version 2.0, while Origin software was used for graphical representation and statistical evaluation.

## Figures and Tables

**Table 1:** Hydrodynamic radii of innate hydrogels and bioactive hydrogels of each respective honey at 37 °C across the 10^0^–10^4^ size range. Distribution range depicts the average of five independent samples for each formulation.

	Z-Average (nm)	Polydispersity Index (PI)	Peak 1 Mean (nm)	Peak 2 Mean (nm)
50% PF127	24.5 ± 1.2	0.161 ± 0.015	28.98 ± 1.4	N/A
40% PF127	18.4 ± 5.5	0.428 ± 0.151	30.4 ± 22.5	N/A
30% PF127	22.5 ± 1.5	0.135 ± 0.025	25.8 ± 2.1	N/A
50% MH	59.98 ± 36.2	0.437 ± 0.122	25.4 ± 2.2	523.1 ± 207.5
40% MH	152.6 ± 57.1	0.398 ± 0.078	21.2 ± 1.1	245.3 ± 74.8
30% MH	159.8 ± 29.9	0.631 ± 0.131	397.05 ± 92.8	23.4 ± 1.4
50% HH	40.4 ± 3.6	0.609 ± 0.068	24.6 ± 0.96	590.1 ± 91.2
40% HH	52.7 ± 21.5	0.702 ± 0.186	136.9 ± 256.3	344.5 ± 189.3
30% HH	84.1 ± 13.4	0.8 ± 0.066	478.5 ± 619.0	791.5 ± 939.4
50% SynH	25.1 ± 1.1	0.284 ± 0.013	24.9 ± 1.4	3242.5 ± 2311.7
40% SynH	27.1 ± 3.3	0.253 ± 0.06	23.4 ± 1.6	1973.3 ± 2253.1
30% SynH	25.5 ± 0.9	0.242 ± 0.021	24.7 ± 1.2	5031.6 ± 125.5

**Table 2:** Formulations of high concentration PF127 and honey.

	PF127 (g)	Innate Honey (g)	Final [PF127] (%(*w*/*w*))	Final [Honey] (%(*w*/*w*))
50% Bioactive PF127	16.67 *	3.443	50	17.215
40% Bioactive PF127	16 **	4	40	20
30% Bioactive PF127	12 **	8	30	40

* 60% Pluronic hydrogel was used to produce a final polymer concentration of 50%; ** 50% Pluronic hydrogel was used to produce a final polymer concentration of 40% and 30%, respectively.

## Data Availability

The raw data supporting the conclusions of this article will be made available by the authors on request.
